# Diversity Analysis and Bioresource Characterization of Halophilic Bacteria Isolated from a South African Saltpan

**DOI:** 10.3390/molecules22040657

**Published:** 2017-04-20

**Authors:** Ramganesh Selvarajan, Timothy Sibanda, Memory Tekere, Hlengilizwe Nyoni, Stephen Meddows-Taylor

**Affiliations:** 1 Department of Environmental Sciences, College of Agriculture and Environmental Sciences, UNISA Science Campus, P.O. Box X6, Florida 1710, South Africa; timsibanda@gmail.com (T.S.); Tekerm@unisa.ac.za (M.T.); 2 Department of Nanotechnology and Water Sustainability, College of Science, Engineering and Technology, UNISA Science Campus, P.O. Box X6, Florida 1710, South Africa; nyonih@unisa.ac.za; 3 College of Agriculture and Environmental Sciences Laboratories, UNISA Science Campus, P.O. Box X6, Florida 1710, South Africa; mtayls@unisa.ac.za

**Keywords:** biodiversity, enzymes, biodegradation, secondary metabolites, halophiles

## Abstract

Though intensive research has been channeled towards the biotechnological applications of halophiles and other extremophilic microbes, these studies have not been, by any means, exhaustive. Saline environments still offer a vast diversity of microbes with potential to produce an array of natural products which can only be unlocked by concerted research efforts. In this study, a combination of culture and molecular approaches were employed to characterize halophilic bacteria from saltpan water samples and profile their potential biotechnological applications. Physicochemical analysis of the water samples showed that pH was alkaline (pH 8.8), with a salinity of 12.8%. 16S rRNA gene targeted amplicon analysis produced 10 bacterial phyla constituting of Bacteroidetes (30.57%), *Proteobacteria* (15.27%), *Actinobacteria* (9.05%), *Planctomycetes* (5.52%) and *Cyanobacteria* (3.18%). Eighteen strains were identified using sequencing analysis of the culturable bacterial strains. From these, the strains SP7 and SP9 were positive for cellulase production while the strains SP4, SP8 and SP22 were positive for lipase production. Quantitative enzyme assays showed moderate extracellular cellulase activity (1.95 U/mL) and lipase activity (3.71 U/mL) by the isolate SP9 and SP4 respectively. Further, of the six isolates, the isolate SP9 exhibited exploitable potential in the bioremediation of hydrocarbon pollution as demonstrated by its fairly high activity against benzanthracene (70% DCPIP reduction). Elucidation of the isolates secondary metabolites showed the production of the molecules 2,3-butanediol, hexahydro-3-(2-methylpropyl)pyrrole[1,2*a*]pyrazine-1,4-dione, aziridine, dimethylamine and ethyl acetate (GC-MS) and oxypurinol and 5-hydroxydecanoic acid (LC-MS), particularly by the isolate *Salinivibrio* sp. SP9. Overall, the study showed that the isolated halophiles can produce secondary metabolites with potential industrial and pharmaceutical application.

## 1. Introduction

Extreme environments are characterized by extreme physicochemical conditions which make them inhabitable for higher life forms [1]. However, some microorganisms are adapted to life at different extreme environmental conditions ranging from extremes of temperature, pH, salinity, radiation and pressure [2,3] and are therefore referred to as extremophiles. The study of extreme environments has significantly unlocked our understanding of extremophilic microbial diversity to some extent by yielding novel taxa from uncultured sequence data [4,5]. Among the extremophiles, thermophiles are the most extensively studied for biotechnological applications [6,7,8,9]; with much less attention being paid to halophilic microbes to explore their roles in biotechnological processes of commercial interest.

Halophiles (salt-loving) are an important group of microorganisms that require salt (NaCl) for growth, and they can be found in all three domains of life, including the Archaea, Bacteria and Eukarya [10]. They have been isolated from different saline habitats such as natural brines, artificial solar salterns, hyper saline lakes, submarine pools and deep salt mines [11,12]. Halophiles are classified in to two groups, moderate and extreme halophiles [13], which have largely undergone different physiological adaptive mechanisms to survive under high salt concentration [10], making them or their products suitable for application in many industrial processes where conditions are saline.

Culture-dependent studies have been done to isolate halophiles from different hypersaline habitats ranging from solar salterns to deep salt mines [14,15,16,17]. However, only a minor fraction of the existing halophile diversity has been explored, largely for enzyme production and other applications like production of bioactive compounds and compatible solutes that are useful as stabilizers for biomolecules or stress-protective agents [11]. Other recent studies have revealed that secondary metabolites of halophilic microbes contain a variety of bioactive compounds like lipopeptides, polypeptides, polyketides, isocoumarins and macrolactins [18,19,20]. These products are in high demand in the pharmaceutical industry where they are used as an antidote to a variety of plant, animal and human pathogens as well as the replacement of some chemical pesticides. At the same time, the advent of new molecular methods like next generation sequencing has revolutionized our understanding of microbial ecology and discovery of novel genes [21]. Further, it has opened up opportunities for the discovery of novel organisms and the exploration of the distribution and roles of organisms in extreme environments [22]. The microbes that undergo natural attenuation in order to adapt to extreme environments could be sources of biotechnologically indispensable molecules with a wide range of applications ranging from producing industrially important enzymes, to bioremediation and other commercial products.

Solar saltpans are found all around the world and provide ideal settings for halophilic and halotolerant microbes [23]. Most of these pans are rich in salts and are often covered with calcium rich caliche, dolomite and marl [24]. To our knowledge, few studies have been reported on the microbial diversity of South African salt pans [25,26], and certainly none of them have reported on the biotechnological relevance of halophiles from these locations. The aim of this study, therefore, was to target both culture independent (to understand the microbial diversity) and culture dependent bacterial isolates from salt pan water samples collected from the Free State Province of South Africa and to determine their ability to produce some target enzymes as well as other secondary metabolites, previously known or unknown, and create a metabolic molecular fingerprint.

## 2. Results

### 2.1. Physicochemical Variables

Table 1 summarizes the physicochemical variables of the saltpan water samples. The examined saltpan water samples were alkaline (pH 8.8). Salinity of the water samples was 12.8%. Sodium (42.6 g/L) and chloride (75.8 g/L) were the most abundant ions followed by calcium (1028 mg/L). The levels of most elemental constituents were below detection limit, with the exception of strontium (31 mg/L), selenium (0.58 mg/L) and boron (0.22 mg/L). The amount of total sulphur (1925 mg/L) was much higher than the amount of total iron (1.51 mg/L).

### 2.2. Culture Independent Analysis

**Figure 1:**
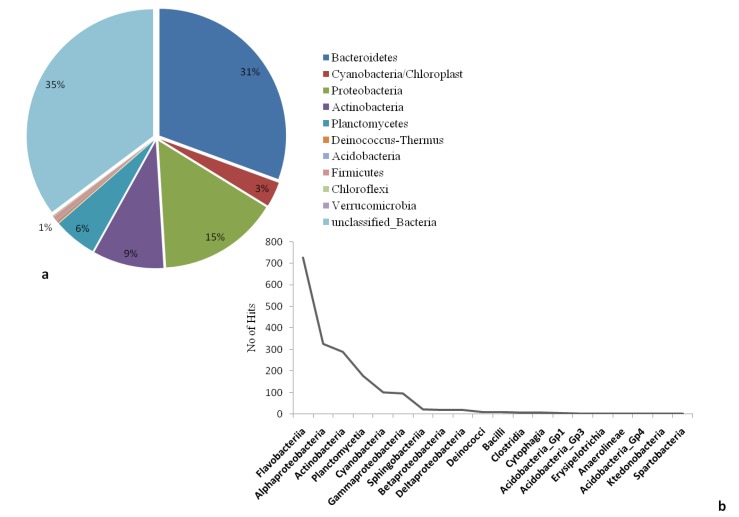
(**a**) Relative abundance and diversity of bacterial phylum detected in the Salt pan water with sequences of the variable region V1–3 of the 16S rRNA genes (**b**) the taxonomic abundances of classes from the most abundant to least abundant.

The microbial community structure and species richness of the collected water samples was analysed using targeted 16S rRNA amplicon sequencing. A total of 3459 sequences were obtained with a mean sequence length of 266 ± 54.8 (Table S1). RDP classifier tool was used to classify the reads based on specific phylogenic bacterial taxa. In total, 10 bacterial phyla were observed excluding unclassified sequences (derived from bacteria). In terms of their abundance; the highest proportion was recorded for unclassified bacterial sequences (35.22%), followed by *Bacteroidetes* (30.57%), *Proteobacteria* (15.27%), *Actinobacteria* (9.05%), *Planctomycetes* (5.52%) and *Cyanobacteria* (3.18%). Representatives of *Firmicutes*, *Deinococcus-Thermus*, *Acidobacteria*, *Chloroflexi* and *Verrucomicrobia* constituted a relatively small percentage of the entire classified bacterial population (Figure 1a). Within the dominant phylum (*Bacteroidetes*), the most abundant class was *Falvobacteria* (40.13%), whereas the other classes such as *Sphingobacteria* (1.22%) and *Cytophagia* (0.28%) occurred in very low percentages. In the phylum *Proteobacteria*, four classes were recorded namely: *Alphaproteobacteria* (17.97%), *Gammaproteobacteria* (5.19%), *Betaproteobacteria* (1.05%) and *Deltaproteobacteria* (1.05%). Similarly, other classes including *Actinobacteria* (15.87%), *Planctomycetes* (9.73%) and *Cyanobacteria* (5.58%) were also recorded in significant proportions (Table S2). The taxonomic abundances of classes from the most abundant to least abundant are presented in Figure 1b. The classification of total reads into lower taxonomic levels revealed extremely diverse bacterial communities in collected samples, with up to 30 orders, 56 families and 64 genera being detected (Table S3).

### 2.3. Culture Dependent Analysis

**Figure 2:**
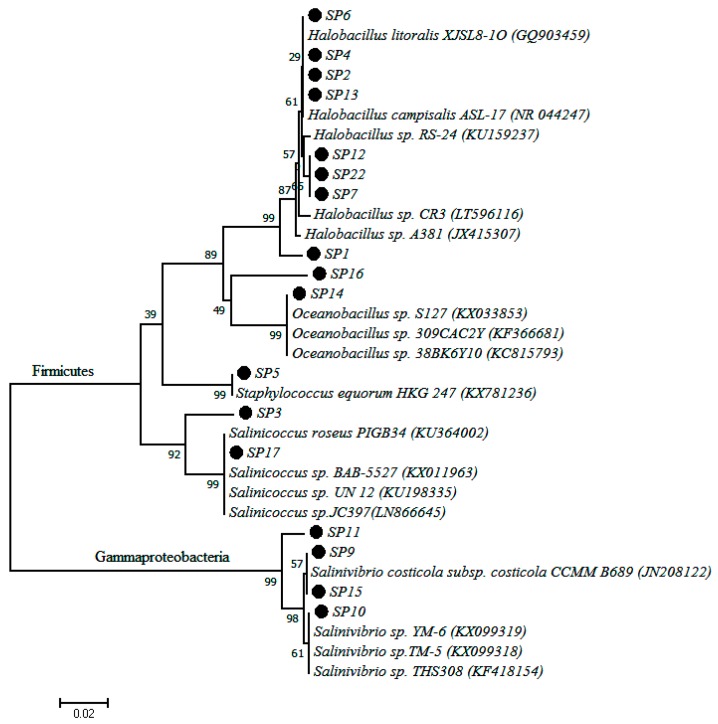
Phylogenetic tree based on 16S rDNA gene sequences obtained by the Maximum Likelihood method showing the phylogenetic relationship among the 18 bacterial isolates of this study (dotted with code names) and related bacteria.

A total of 18 different pure isolates were obtained from the collected water samples. Phylogenetic comparison of PCR amplified 16S rDNA sequence data of each isolate with the database of known species using the NCBI server revealed that bacteria of the genus *Halobacillus* were the most prevalent, with 44.44% of the isolates falling into this genus. This was followed by the genus *Salinivibrio* and *Saliniococcus* each of which comprised with 22.2% and 11.1% of the total isolates, respectively. Other isolates belonging to the genera *Oceanobacillus*, *Thalassobacillus*, *Bacillus* and *Staphylococcus* were also recorded. The pairwise nucleotide sequence similarity of all isolates ranged from 99% to 100%. All the bacterial isolate codes, sequence length, percentage similarity to closest matching strains and accession numbers are given in Table 2. In order to evaluate their phylogenetic positions, the 16S rRNA gene sequence of each strain was analyzed and a phylogenetic tree was constructed by using Mega 6 software. The phylogenetic tree showed that isolates belong to two major groups, *Firmicutes* and *Gammaproteobacteria* (Figure 2). The Firmicutes group comprised the major genus of the isolated strains while the genus *Salinivibrio* was the only representative of *Gammaproteobacteria*. The phylogenetic tree also confirms that all isolates of the genus *Salinivibrio* were distinctly different from the rest of the isolates and their closest relatives.

### 2.4. Enzyme Assay

Out of 18 isolates, only two strains *Halobacillusalkaliphilus* SP7 and *Salinivibrio* sp. SP9 showed a clear zone indicating the production of cellulase after flooding with Gram’s iodine. In contrast, no clearance zone was observed in other strains (Table 3). A quantitative cellulase assay was carried out spectrophotometrically by using microcrystalline cellulose as substrate and cell free spent medium as enzyme source as described. Halophilic bacterial isolate *Salinivibrio* sp. SP9 showed highest extracellular cellulase activity of 1.95 U/mL whereas the strain *Halobacillus alkaliphilus* SP7 recorded 0.23 U/mL cellulase activities. On the other hand, screening for lipase production showed that only isolate *Halobacillus* sp. SP4, *Halobacillus alkaliphilus* SP8 and *Halobacillus alkaliphilus* SP22 could hydrolyze olive oil. A quantitative lipase assay was carried out for the positive strains spectrophotometrically by using phenyl acetate. The maximum lipase activity of 3.71 U/mL was observed in the strain *Halobacillus* sp. SP4, whereas the lipase activity of *Halobacillus alkaliphilus* SP8 and SP22 was 1.42 and 1.72 U/mL, respectively.

### 2.5. Hydrocarbon Degradation Assay

All the isolates from the saltpan were subjected to hydrocarbon screening. Out of 18 isolates, six strains including *Halobacillus* sp. SP4, *Staphylococcus* sp. SP5, *Halobacillus* sp. SP6, *Salinivibrio* sp. SP9, *Salinicoccus* sp. SP17 and *Halobacillus alkaliphilus* SP22 were able to utilize the tested hydrocarbon as a carbon source during preliminary screening (Table 3). The positive isolates during hydrocarbon screening were further applied to hydrocarbon degradation assays using three classes of hydrocarbons, namely benzanthracene, naphthalene and diesel fuel using 2,6-dichlorophenol indophenol (DCPIP) as the indicator. During the assay, optical density (OD) was taken at 600 nm at regular intervals and the percentage of significant reduction was obtained using the following equation:

DCPIP reduction (%) = (Intial O.D – Final O.D) − Intial O.D × 100%
(1)

Isolate *Salinivibrio* sp. SP9 showed the highest potential of the six test isolates to metabolize all three hydrocarbon classes, with the highest activity against benzanthracene (70% DCPIP reduction).

**Figure 3:**
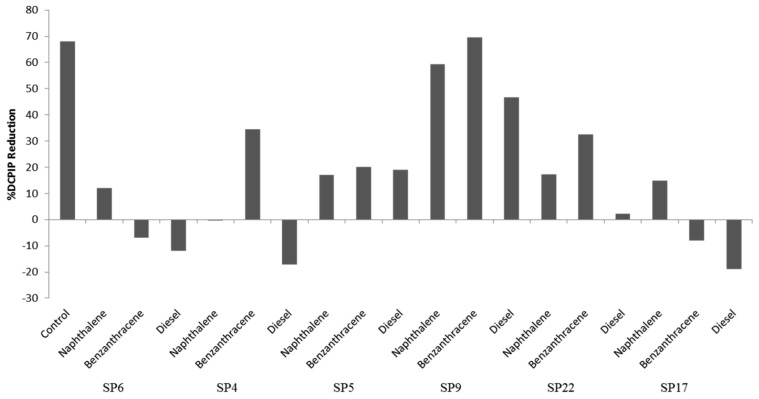
Percent reduction of DCPIP during hydrolysis of hydrocarbons by six bacterial isolates.

Isolate *Halobacillus alkaliphilus* SP22 showed 35% reduction of DCPIP when using benzanthracene and 25% when metabolizing naphthalene. Isolate *Staphylococcus* sp. SP5 is a halotolerant which showed approximately 20% reduction of DCPIP for all three hydrocarbon classes. Isolates *Halobacillus* sp. SP6 and *Salinicoccus* sp. SP17 showed 15% and 20% DCPIP reduction when metabolizing naphthalene, even though they could not metabolize the other two classes of hydrocarbons (Figure 3).

### 2.6. Secondary Metabolites of Halophilic Bacteria

**Figure 4:**
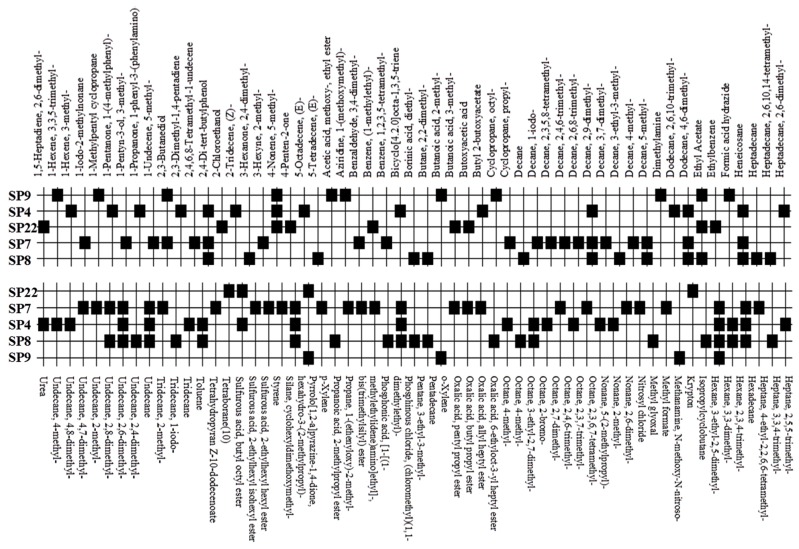
A map of secondary metabolites produced by bacterial isolates from saltpan as detected by GC-MS.

Five bacterial isolates (SP4, SP7, SP8, SP9 and SP22) which had tested positive when screened for production of either lipase or cellulase were taken for both GC-MS and UHPLC-QToF-MS analysis of their secondary metabolites. GC-MS secondary metabolite elucidation was able to identify 113 different compounds. These compounds were identified by comparison of their mass spectra with the Wiley spectral library based on their molecular weight and retention time. To ensure a higher degree of accuracy, the minimum similarity cut-off match for the compound was fixed at 700. All the compounds were constructed into a map (Figure 4) through multivariate seriation analysis using Paleontological Statistical software v3.13 [27]. Due to the vast number of compounds that were produced by the bacterial isolates, the data was subjected to principal component analysis (PCA) and the compounds which fell off the nucleus were chosen for further analysis and discussion. Table 4 represents the structure and potential documented uses of some halophilic bacterial secondary metabolites identified by GC-MS. Metabolite concentrations were, however, not quantified since the analysis was largely a screening test which made it difficult to incorporate standards for the purposes of quantification. Isolate *Salinivibrio* sp. SP9 produced the most number of industrially and pharmaceutically important molecules which included 2,3-butane-diol, hexahydro-3-(2-methylpropyl)pyrrolo[1,2*a*]pyrazine-1,4-dione, aziridine, dimethylamine and ethyl acetate. The strain *Halobacillus alkaliphilus* SP7 was considered as the second most important producer of some metabolites like styrene and 2,3-butanediol in this study, as well as some bioactive molecules such as phosphonic acid and cyclohexyl(dimethoxy)methylsilane. Similarly, the bioactive compounds borinic acid and methylglyoxal could only be produced by the isolate *Halobacillusalkaliphilus* SP8.

**Figure 5:**
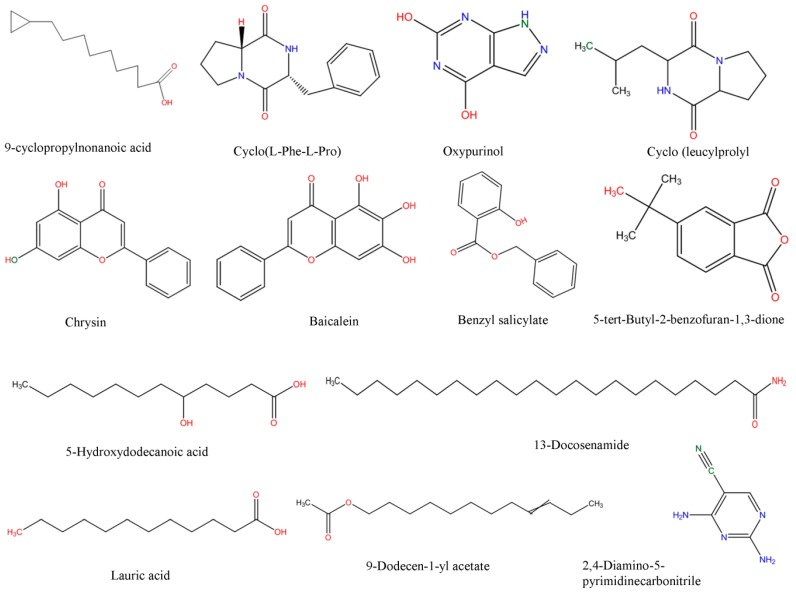
Structural elucidation of halophilic bacterial secondary metabolites identified using UHPLC-MS.

Polar secondary metabolites were analyzed using UHPLC-QToF-MS. Elucidation of these compounds was done using different Bruker software programs such as Data Analysis v4.3 and Profile Analysis v2.0. To analyze the LC-MS determined-compounds, the total dataset containing all 2304 extracted compounds detected within the *m*/*z* range of 100–1700 was first analysed using PCA to identify any outliers and assess any groupings or trends. Principal component 1 (PC1) showed that isolate *Salinivibrio* sp. SP9 produced a different pattern of metabolites which were significantly different from those of other isolates. PC2, though, further showed that SP4 was metabolically different to the other isolates. PCA loadings showed that all the isolates could produce the compounds 9-cyclopropylnonanoic acid, cyclo-(l-Pro-l-Phe), cyclo(l-Leu-l-Pro), chrysin, baicalein, benzyl salicylate, 5-*tert*-butyl-2-benzofuran-1,3-dione, 13-docosenamide, lauric acid, 9-dodecen-1-yl acetate and 2,4-diamino-5-pyrimidinecarbonitrile while *Salinivibrio* sp. SP9 was the only isolate which could produce oxypurinol and 5-hydroxydecanoic acid. Structural elucidation of the compounds was done using online libraries including KEGG, PubChem and Chemspider. The molecular structures of the selected compounds, which were sketched using the fragmentation explorer of the Compass data analysis software, are shown in Figure 5.

## 3. Discussion

Microbes are the most dominant successful organisms, tolerating a wide range of physicochemical stresses in almost all ecosystems on the planet [44]. However, it is now commonly accepted that more than 95% of microbes exist in environments that have not yet been explored [45]. Extreme environments like hypersaline habitats harbor functional and taxonomical diversified microbial communities [46]. Many microorganisms that are adapted to life at high-salt concentrations are a reservoir for a number of molecules with potential for commercial interest [11]. Currently, the challenge besetting the study of biological molecules produced by halophiles is that their potential applications may not be well known, or even unknown [4].

Hypersaline habitats show a great variability in their physicochemical variables including pH, salinity and ionic compositions [47]. For example, the Big Soda Lake in western USA is highly alkaline (pH 9–10) with salinity of about 10%, while the Great Salt Lake has a salinity of over 20% and a pH of around 7 [48]. As in the studies just cited, our samples had a pH of 8.84 but a salinity of 12.8%. According to Ollivier et al. [47], some hypersaline ecosystems are dominated by Na^+^ and Cl^−^ ions, which was also observed in this study (Table 1). The amount of total sulphur (1925 mg/L) in our sample was much higher than the recorded levels for the Dead Sea (480 mg/L) [47]. Sulphur plays some important role in the electron transfer mechanism involved in precipitation of organic matter. As reported, the dynamics of microbial populations always depend on the physico chemical variables of the particular environment [49].

Hypersaline environments such as saltpans have widely been reported to contain a diversified microbial population [16,23,50,51], of which the haloarchaea is by far the most dominant and successful, boasting over 19 identified genera and over 57 described species [52,53]. However, the microbial biodiversity inhabiting South African saltpans remains poorly understood. To our knowledge, this study presents the first report of bacterial diversity from Saltpan water in South Africa. Using next generation sequencing, our study revealed that a total of 10 phyla (Figure 1a) containing 64 genera were observed in the collected water samples. Similar observations have been reported in soda ash concentration pond samples [54]. Our retrieved bacterial sequences show that the saltpan harbors populations of sulphur reducing/oxidizing bacteria belonging to the unique order *Caulobacterales*, *Hydrogenophilales*, *Desulfovibrionales*, *Acidimicrobiales* and *Ktedonobacterales* (data not shown). Occurrence of these members was likely influenced by the presence of iron and sulphur in the saltpan water (Table 1). Some researchers have previously reported that diversity of cyanobacteria in salt pans was generally low [55,56]. Cyanobacteria in the current study averaged about 3.18% of the total reads, a finding which seemed to agree with previous researchers’ observations. Importantly though, cyanobacteria are responsible for active nitrogen cycle in saline systems [54]. Interestingly, the order *Halanaerobiales* was recorded in this study which is affiliated to low G+C branch of the *Firmicutes*. Species of this order metabolize sulfur in a different manner by using selenate and arsenate as electron acceptors [57]. Another interesting observation from this study was the percentage of unclassified bacterial reads which was higher than classified reads, suggesting that this saltpan may contain potentially novel microbial species.

In culture dependent studies, the most common bacteria isolated from saltpans belong to clades of Alphaproteobacteria, Gammaproteobacteria and Firmicutes [15]. In this study, a partial 16S rRNA gene sequence resulted in the identification of eighteen strains from the saltpan; which belonged to two major phyla, the Firmicutes and Gammaproteobacteria. This observation was in agreement with the findings of Vahed et al. [58] who also reported dominance by the same phyla in water samples of a saline lake in Iran. Some surveys of the bacterial diversity of saltpans resulted in the discovery of novel halophilic bacteria like Salinibacterruber and Salicolamarasensis, which can survive up to 30% salt concentrations [10,59]. Halophiles are rich sources of halozymes like lipase, protease, cellulase and xylanase, which can tolerate high saline conditions, with potential application in industrial processes where conditions are saline [23]. In the present study, five halophilic bacterial isolates were found to produce cellulase and lipase by utilizing CMC and olive oil respectively (Table 3). Sánchez-Porro et al. [16] reported the ability of halophilic strains isolated from salterns in Spain to produce different extracellular enzymes (protease, lipase, DNase and pullulanase). Similarly, Govender et al. [60] also reported that halophilic bacteria from solar salterns in Botswana produced different hydrolytic enzymes like cellulase, mannanase and xylanase. In our study, the gram-positive genus *Halobacillus* (SP4, SP7, SP8 and SP22) was the dominant producer of hydrolytic enzymes (cellulase and lipase), a finding which concurred with previous reports [15,16]. However, our findings were contrary to the findings of Babavalian et al. [61] who reported that lipase enzyme was more frequently produced by gram negative than gram positive bacteria. In terms of enzyme activity, the strain *Salinivibrio* sp. SP9 showed highest cellulase activity of 1.95 U/mL, which was closer to the report of Wang et al. [62] who observed a similar range of activity in *Salinivibrio* sp. strain NTU-05 isolated from Szutsausaltern. The strain *Halobacillus* sp. SP4 recorded the highest lipase activity of 3.71 U/mL compared to the other strains, with olive oil as the substrate. A similar range of activity (3.41 U/mL) has been reported from the strain *B. vallismortis* BCCS 007 isolated from the Maharla salt lake in South Iran [63]. Enzyme production was targeted in this study because enzyme based technologies for the synthesis of novel compounds is on the increase [64]. Lipases are arguably one of the enzymes with the broadest spectrum of applications ranging from food industry, oil and fat industry, detergent industry, leather industry, pulp and paper industry, detergent industry, cosmetic industry, biodiesel production as well as in organic chemistry [64]. Because of their broad applications in enzyme technology, research aimed at discovering microbial producers of lipases and other enzymes from previously unexplored environments should be encouraged. Petroleum and natural gas reservoirs frequently contain hypersaline waters. The search for ideal microorganisms for bioremediation of saline ecosystems contaminated with hydrocarbons is in progress all over the world [65]. Fathepure [66] explained that polycyclic aromatic hydrocarbons (PAHs) are abundant in many oily and saline environments posing a high environmental risk factor due to their toxic, mutagenic and carcinogenic properties. However, information on the ability of halophiles to treat hypersaline environments contaminated with aromatics is limited [67]. The potential of halophilic and halotolerant bacteria to degrade hydrocarbons (benzanthracene, naphthalene and diesel fuel) was profiled in this study using DCPIP as the indicator (Table 3). DCPIP is an electron acceptor that becomes reduced (decolorized) when redox reactions occur, in this case when NADH is converted to NAD^+^ during microbial degradation of hydrocarbons [68]. The isolate *Salinivibrio* sp. SP9 showed the highest hydrocarbon-degrading activity (Figure 3) against benzanthracene (70% DCPIP reduction) followed by *Halobacillus alkaliphilus* SP22 (35% DCPIP reduction). To our knowledge, this is the first report linking the *Salinovibrio* genus to hydrocarbon degradation activity as previous studies have only documented the biodegradation of benzanthracene by some bacteria of the genera *Alcaligenes*, *Stenotrophomonas*, *Sphingomonas* and *Pseudomonas* [69]. Interestingly, the isolate *Staphylococcus* sp. SP5 is a halotolerant which showed approximately 20% of DCPIP reduction for all three hydrocarbon classes, a finding that agrees with the observations of Mujahid et al. [70] who reported aromatic hydrocarbon degradation by *Staphylococcus* sp. SA061. These reports clearly demonstrate the potential of both halophilic and halotolerant bacteria from saltpans to degrade PAHs under hypersaline conditions.

Besides degrading hydrocarbons, halophilic bacteria have the ability to produce a variety of bioactive compounds due to adverse environmental habitats which are not encountered by their terrestrial counterparts [19]. Isolates *Salinivibrio* sp. SP9 and *Halobacillus alkaliphilus* SP22 could produce the compound hexahydro-3-(2-methylpropyl)pyrrolo[1,2*a*]pyrazine-1,4-dione which is commonly used as a broad spectrum antibiotic for treating some bacterial, fungal and nematode infections [33]. This compound has also been documented to have anti-cancer properties [34]. The compound aziridine is a highly valuable heterocyclic compound which was produced by the strain *Salinivibrio* sp. SP9. This compound is widely used in drug synthesis and has many therapeutic uses [41]. Trippier and McGuigan [32] reported a novel class of borinic acid which exhibits a lot of medicinal applications including anticancer and anti-microbial properties. Microorganisms which produce anticancer drugs deserve increased attention given the growing global incidence of this disease, and the discomfort of the current chemotherapeutic methods of treating it. In this study, strain SP8 produced borinic acid which has potential application in the treatment of dermatological diseases including acne and atopic dermatitis [31]. In addition, the strain *Halobacillus alkaliphilus* SP8 produced the compound methylglyoxal which was previously reported in Manuka honey as having the ability to control the multidrug-resistant *Pseudomonas aeruginosa* [40] by inhibiting the protein synthesis of the bacterium. Methylglyoxal is also used in healing diabetic ulcers [39]. Finally, isolate *Halobacillus alkaliphilus* SP7 produced phosphonic acid which is used as a generic hapten in the production of antibodies against a group of organophosphorus pesticides [38], besides being widely marketed as a fungicide in commercial fertilizers [37]. In addition, the same strain could produce cyclohexyl(dimethoxy)methylsilane which can potentially be used as an important component of polymeric substances for filling dental cavities [35]. A quick glance at Table 4 shows that most of the compounds produced by the halophilic isolates are bioactive ingredients of commercial antimicrobials. However, some of the isolates could also produce some products with potential for industrial applications. Isolate SP9 for instance could produce dimethylamines and 2,3 butane diol which is used as a precursor for the production of dimethyl acetamide (DMAC), choline chloride (CC) and dimethyl formamide (DMF) [42] and a range of chemicals products [30]. Also, *Salinivibrio* sp. SP9 produces ethyl acetate which is used as flavoring compound in the wine industry [43].

Most of the compounds (Figure 5) selected from UHPLC-QToF-MS analysis were produced by all halophilic bacterial isolates except oxypurinol and 5-hydroxydecanoic acid which was produced only by *Salinivibrio* sp. SP9. The compound oxypurinol is the only active metabolite of xanthine oxidase inhibitor, used for the treatment of gout and congestive heart failure [71]. The compound 5-hydroxydecanoic acid (lipopolysaccharide) has previously been isolated as a metabolite of *Salmonella* sp. and reported to protect the retina against light-induced injury [72]. The compounds chrysin (5,7-dihydroxyflavone) and baicalein (5,6,7-trihydroxyflavone) which were produced by all isolates are known natural flavonoids found in many plant extracts that are commonly used as wound healing, skin protective, anti-tumor and anti-cancer medicines [73,74]. Microbial production of dipeptide has previously been reported [19,20]. The compounds cyclo-(l-Pro-l-Phe) and cyclo(l-Leu-l-Pro) (produced by all isolates in this study) are known cyclic dipeptides used as scaffolds for drug synthesis besides their use as antiviral, antifungal and anti-tumor drugs [75,76]. One of the identified compounds, benzyl salicylate, is a known salicylic acid benzyl ester which is used as an antifungal agent [77]. Other identified compounds including 5-*tert*-butyl-2-benzofuran-1,3-dione,9-dodecen-1-yl acetate and 2,4-diamino-5-pyrimidine carbonitrile have no known uses, as do a host of other compounds which any of the online libraries like Chemspider, PubChem and KEGG could detect. This clearly indicates that exploration of novel microbial metabolites still has a long way to go. The documentation of salt tolerant natural product chemistry must be actively pursued to develop unique compounds in the field of biomedical and pharmaceutical industries. In a bid to increase the success rate in the identification of novel microbial secondary metabolites, the use of GC-MS and UHPLC-QToF-MS in chemical profiling of crude fermentation extracts can be a very useful tool for assessing the chemical novelty of the crude extracts by comparing the mass spectra to in-built and online compound libraries.

## 4. Materials and Methods

### 4.1. Description of Study Area, Sample Collection and Bacterial Isolation

Soutpan (Salt Pan) is a small salt mining town in South Africa just north of Bloemfontein, near the Soetdoring Nature Reserve. According to Day [78] the greatest concentrations of salt pan befalls in the north-western Cape and the western Transvaal and Orange Free State in South Africa. The main industrial activities in the area are salt mining and farming. Grab water samples were collected for both water chemistry and microbial analysis during the summer season (November 2015) from the same saltpan (−28°75′26.93 S, 26°04′77.86 E). Physicochemical parameters temperature, pH, conductivity, salinity, total dissolved solids (TDS), ORP, and dissolved oxygen (DO) were measured and recorded on site during sampling using a multi-parameter meter (Hanna Instruments PTY Ltd., Johannesburg, South Africa). The samples were immediately stored in cooler boxes containing ice and transported to the laboratory at UNISA Science Campus for analysis within 12 h of collection. One hundred microliter (100 µL) aliquots of the water samples were spread-plated on minimal salt agar made by dissolving, in g/L; meat extract (3), casein peptone (5), agar (15), NaCl (3%, 9% and 14%), and on HALO medium having (g/L): NaH_2_C_3_H_5_O (COO)_3_ (10), Na_2_SO_3_ (10), C_24_H_39_NaO_5_ (3), C_12_H_22_O_11_ (20), FeC_6_H_5_O_7_ (1), KH_2_PO_4_ (2), MgSO_4_·7H_2_O (5), NaCl (1%, 3% and 5%). Culture medium pH was adjusted to 7.0 ± 0.2 before autoclaving. The plates were incubated at 30 °C for 48 h. The resultant (mixed) cultures were separated and purified by sub-culturing onto nutrient agar plates supplemented with 1 M NaCl, until axenic cultures were obtained.

### 4.2. DNA Extraction and Sequence Analysis

For 16S rRNA gene targeted amplicon analysis, water samples were filtered using 0.45 µm pore-sized filter membranes which were then subjected to total DNA extraction using a Quick *g*-DNA Extraction Kit^TM^ (Zymo Research Corporation, Orange, CA, USA) following the manufacture’s protocol. Total DNA was then quantified using a NanoDrop spectrophotometer (Nanodrop 2000, Thermo Scientific, Kyoto, Japan). Polymerase chain reaction (PCR) was performed on the extracted DNA samples using the universal bacterial primers 27F and 518R targeting the variable region V1-V3 of the 16S ribosomal DNA. The thermal profile consisted of a first denaturing step at 95 °C for 5 min, followed by 32 amplification cycles of denaturation at 95 °C for 30 s, annealing at 52 °C for 30 s, elongation at 72 °C for 1 min and a final extension step at 72 °C for 10 min. PCR amplicons were purified using a purification kit (Qiagen, Valencia, CA, USA). Following the purification step, the pooled PCR products were sequenced on the GS-FLX-Titanium series 454/Roche by Inqaba Biotechnology (Pretoria, South Africa). The raw sequence data were processed in RDP pipeline following the method of Wang et al. [79]. Chimeric sequences were removed from ribosomal sequences using UCHIME [80] and non-chimeric rRNA sequences were analyzed using RDP classifier with an 80% confidence threshold. The resulting classifier sequences were uploaded on the Align tool and finally the cluster files were generated with complete linkage clustering tools. Genetic distance was determined and sequences were clustered into operational taxonomic units (OTUs) using MOTHUR. Finally, the amplicons obtained in this study were submitted to DNA Data Bank of Japan (DDBJ) to obtain the accession number (PRJDB5304).

Sanger sequence analysis was done for pure isolates. DNA was isolated from each culture using a Quick *g*-DNA extraction kit (Zymo Research Corporation) followed by PCR amplification using 16S universal bacterial primers (27F and 1492R) and the thermal profile described above. The PCR amplicons were purified and sent to Inqaba Biotech (Pretoria, South Africa) for sequence analysis. The resultant sequences were subjected to BLAST analysis to compare the identity of the isolates. Phylogenetic analysis was done using Molecular Evolutionary Genetic Analysis v6 (MEGA6) software (Center for Evolutionary Medicine and Informatics, Tempe, AZ, USA). Finally, all the sequences obtained in this study were submitted to GenBank to obtain the accession numbers (Table 2).

### 4.3. Lipase Assay

The halophilic bacterial isolates were screened for the production of lipase in rhodamine-olive oil-agar medium following the method of Kumar et al. [81] with slight modifications. The agar medium contained, in g/L, agar-agar 20, MgSO_4_ 0.2, CaCl_2_ 0.02, KH_2_PO_4_ 1.0, K2HPO_4_ 1.0, NH_4_NO_3_ 1.0, FeCl_3_ 1.0, and yeast extract. The medium was supplemented with 1 M NaCl, autoclaved and cooled to about 50 °C after which 31.25 mL of olive oil and 10 mL of rhodamine B solution was added. The plates were inoculated with axenic bacterial cultures and incubated for 48 h at 37 °C. Utilization of olive oil was identified under UV by formation of orange fluorescent halos around bacterial colonies due to the hydrolysis of substrate. Lipase activity was determined spectrophotometrically by following the protocol given by Abd-El Hakeem et al. [82]. One lipase unit (U) was defined as the amount of enzyme that liberates 1 µmol *p*-nitrophenol/min under the described assay conditions.

### 4.4. Cellulase Assay

For cellulase screening, minimal salt medium containing 0.2% (*w*/*v*) carboxymethyl cellulose sodium salt (CMC) and 1% agar supplemented with 1 M NaCl was prepared. Spot inoculation was then done using axenic cultures and incubated at 30 °C for 48 h. Positive strains showed hydrolysis zones when the plates were visualized by formation of halos after flooding with 0.1% Congo red stain (Glass World, Johannesburg, South Africa) followed by destaining with 1 M NaCl. Cellulase activity was determined spectrophotometrically by following the method given by Worthington [83]. One unit was defined as the amount of enzyme needed to liberate 1.0 μmol of glucose from cellulose in one hour at pH 5.0 at 37 °C after 2 h incubation time.

### 4.5. Hydrocarbon Degradation Assay

A modified protocol of Um et al. [84] was used to test the isolates for the capacity to degrade hydrocarbons. Minimal Salt Medium (MSM) supplemented with 1.25% (*w*/*v*) of agar was used as ‘bottom agar’. The bottom agar was then overlaid with 100 µL solution containing a mixture of naphthalene and benzanthracene in methanol. The polyaromatic hydrocarbons (PAHs) solution was evenly spread over the agar surface and evaporated to leave a visible thin white layer of PAHs on the surface of the bottom agar. Thereafter, 100 µL of each bacterial isolate was mixed with molten ‘top agar’ medium containing 0.5% agar and immediately poured on petri plates containing bottom agar. The plates were then swirled gently, left to solidify and incubated at 25 °C. The plates were observed daily for 7 days for the presence of growth and clear zones.

The isolates which were able show to clear halos around colonies were selected for further hydrocarbon degrading assays following the slightly modified method of Oliveira et al. [85]. The positive isolates were grown in fermentation broth containing minimal salt medium supplemented with glucose (10 g/L) and yeast extract (1.0 g/L) and incubated at 25 °C for 72 h with shaking at 120 rpm. After incubation, the culture was centrifuged at 10,000× *g* for 10 min. The pellet was re-suspended in phosphate buffer and again centrifuged to remove all culture medium residues. The pellet was finally re-suspended in phosphate buffer and the OD adjusted to McFarland 0.5. The assay was carried out by using DCPIP as an indicator in a sterile 96-well microtiter plate. Each well contained 20 µL of the isolate, 168 µL of minimal salt medium, 12 µL of DCPIP and 2 µL of hydrocarbon (naphthalene, benzanthracene and diesel) in turn. In addition to the test wells, both positive (10% glucose) and negative controls were added. The plates were incubated at 30 °C and the readings were taken at 600_OD_ in a photometer after 24, 48 and 72 h of incubation, respectively.

### 4.6. Analysis of Bacterial Secondary Metabolites

Bacterial isolates which were positive for any or all of the above screens were grown in minimal salt medium supplemented with olive oil, 1% CMC-Na salt and 1 M NaCl for 14 days in a shaking incubator at 30 °C. After incubation, bacterial cells were separated by high speed centrifugation at 4 °C. The supernatant was split into 2 fractions, one of which was used for enzyme assay (cellulase and lipase) while the other was subjected to solvent extraction of secondary metabolites. A mixture of chloroform and methanol (1:1, *v*/*v*) was added to the supernatant in a 1:1 (*v*/*v*) ratio and shaken at 120 rpm and 25 °C for 18 h. The solvent fraction was then separated from the aqueous phase and evaporated to dryness in vacuo at 80 °C. The residue was re-constituted in a mixture of 1:1 (*v*/*v*) acetonitrile and hexane. The acetonitrile fraction containing polar compounds was analyzed using UHPLC-QToF-MS while the hexane fraction containing non-polar compounds was analyzed using GC-MS.

For GC-MS analysis of secondary metabolites, an HP-5MS fused silica capillary column (30 m, 0.25 mm i.d., 0.25 μm film, cross-linked to 5% phenyl methyl siloxane stationary phase) was used under the following GC conditions: flow rate of mobile phase (Helium) was set at 1 mL/min, ionization energy 70 eV, injection volume 2 µL, split ratio 10:1, injection temperature 250 °C, ion source temperature 200 °C, oven temperature 110 °C (isothermal at 2 min) with increase of 10 °C/min to 200 °C then 5 °C/min to 280 °C with 9 min isothermal at 280 °C. The MS conditions were ionization energy 70 eV, scan interval 0.5 s, fragments 45 to 450 kD and solvent delay 0 to 2 min. Final results were compared by using the Wiley spectral library search programme.

For UHPLC-QToF-MS, the samples were analyzed using an Agilent Ultra-high performance liquid chromatography mass spectrophotometer (Compass QToF Series 1.9, Bruker Instrument: Impact II) system. Chromatographic separation was achieved using an Acquity UPLC BEH C18 column 1.7 um, diameter 2.1 × 100 mm (Miscrosep Waters, Johannesburg, South Africa). The mobile phase consisted of formic acid (FA) in water and acetonitrile. The column flow was set at 0.3 mL/min, column oven temp at 35 °C, draw speed at 2 µL/s with a total injection volume of 2 µL. The parameters for the mass spectrometer (MS) were as follows: capillary voltage 4500 V, drying gas 8 L/m, gas temperature 220 °C, ionization energy 4.0 eV, collision energy 7.0 eV, cycle time 0.5 s. Data analysis was done using the Bruker Software (Bruker Compass Data Analysis 4.3, Bruker Daltonik GmbH, Bremen, Germany, 2014). Final results were compared by using National Institute of Standards and Technology (NIST 2005) library.

## 5. Conclusions

In conclusion, the present study reported the halophilic bacterial community structure from a saltpan using a high throughput next generation sequencing approach. The *Bacteroidetes* were the dominant phyla in culture independent studies while in culture dependent studies, members of the *Firmicutes* and *Gammaproteobacteria* were dominant. The existence of unclassified bacterial phylum suggests the possibilities of finding some novel bacteria within the studied site. Culture dependent assays showed the potential of some halophiles to biodegrade hydrocarbons, making them potentially important agents for the bioremediation of hydrocarbon polluted sites. Further, enzymatic assays showed the potential of some strains to produce the enzymes lipase and cellulase, making them potentially important agents for application in industrial processes where saline environments are unavoidable. GC and LC-MS analyses of secondary metabolites showed the potential of the isolated strains to produce products with potential for use mostly as pharmaceuticals.

## Figures and Tables

**Table 1:** Physicochemical parameters of collected water samples.

Parameters		Units	Saltpan (Soutpan)
Physicochemical	Temperature	°C	26.9
	pH	-	8.84
	Dissolved oxygen	mg/L	1.58
	Conductivity	S/m	15.96
	Salinity	%	12.8
	TDS	g/L	102.6
	ORP	mV	96.5
Majority ions Nutrients	Total Iron	mg/L	1.51
	Silicon	mg/L	5.35
	Calcium	mg/L	1028
	Potassium	mg/L	210
	Magnesium	mg/L	206
	Sodium	g/L	42.6
	Chloride	g/L	75.8
Trace Elements	Silver	mg/L	Trace
	Aluminum	mg/L	Trace
	Arsenic	mg/L	Trace
	Boron	mg/L	0.22
	Barium	mg/L	Trace
	Beryllium	mg/L	Trace
	Bismuth	mg/L	Trace
	Molybdenum	mg/L	Trace
	Nickel	mg/L	Trace
	Lead	mg/L	Trace
	Selenium	mg/L	0.58
	Strontium	mg/L	31.0
	Lead	mg/L	Trace
	Tellurium	mg/L	Trace
	Vanadium	mg/L	Trace
	Zinc	mg/L	Trace
Other Parameters	Total Sulphur	mg/L	1925

Measurable quantities of trace elements lower than <0.2 mg/L considered as ‘Trace’.

**Table 2:** Characterization of isolates by isolate codes, sequence length, percentage similarity to closest matching strains and accession numbers.

Isolate Code	Sequence Length (nt)	Closest Similarity	% Similarity	Accession Number
SP1	859	*Thalassobacillusdevorans* strain HME8790	99	KX885465
SP2	888	*Halobacillus* sp. K13	99	KX885460
SP3	744	*Salinicoccushispanicus* strain muz4C4	99	KX885467
SP4	972	*Halobacillus* sp. K13	99	KX885456
SP5	768	*Staphylococcus equorum* NIIST B-509	99	KX885459
SP6	879	*Halobacillus* sp. A381	99	KX885454
SP7	929	*Halobacillusalkaliphilus* strain MGR92	100	KX885457
SP8	876	*Halobacillusalkaliphilus* strain MGR92	100	KX885463
SP9	935	*Salinivibrio* sp. 89Y	100	KX885462
SP10	860	*Salinivibrio* sp. YH4	99	KX885455
SP11	612	*Salinivibriocosticola* partial	99	KX885471
SP12	880	*Halobacillus* sp. K13	99	KX885461
SP13	831	*Halobacillus* sp. A381	99	KX885469
SP14	920	*Oceanobacillus* sp. 309CAC2Y12	100	KX885470
SP15	899	*Salinivibrio* sp. 89Y	100	KX885466
SP16	922	*Bacillus toyonensis* strain L38	100	KX885458
SP17	621	*Salinicoccus* sp. UN 12	99	KX885468
SP22	876	*Halobacillusalkaliphilus* strain MGR92	100	KX885464

**Table 3:** Screening results for hydrocarbon degradation, cellulase and lipase production.

Isolate	Substrate		
	Hydrocarbon	CMC Salt (Cellulase)	Olive Oil (Lipase)
SP1	−	−	−
SP2	−	−	−
SP3	−	−	−
SP4	+	−	+
SP5	+	−	−
SP6	+	−	−
SP7	−	+	−
SP8	−	−	+
SP9	+	+	−
SP10	−	−	−
SP11	−	−	−
SP12	−	−	−
SP13	−	−	−
SP14	−	−	−
SP15	−	−	−
SP16	−	−	−
SP17	+	−	−
SP22	+	−	+

+ positive isolates; − negative isolates.

**Table 4:** Description of structure and uses of some bacterial secondary metabolites identified by GC-MS.

Isolate	Compound Name and Chemical Structure	Potential Known Applications	References
SP7 SP9	2,3 Butane diol 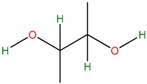	Precursor in the manufacture of a range of chemical products. Antifreeze agent. Synthetic rubber production	[28,29,30]
SP8	Borinic acid 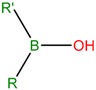	Used in treatment of dermatological diseases including acne and atopic dermatitis	[31,32]
SP22 SP9	Hexahydro-3-(2-methylpropyl)-pyrrolo[1,2*a*]pyrazine-1,4-dione 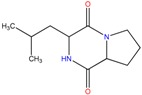	Antibacterial, antifungal, nematicidal and anti-cancer properties. Also, commonly used as broad spectrum antibiotics	[33,34]
SP7	Cyclohexyl(dimethoxy)methylsilane, 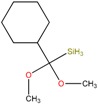	Used to mediate interfacial bonding in mineral reinforced dental polymeric composites.	[35]
SP7	Styrene 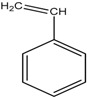	used in the production of plastics and resins	[36]
SP7	Phosphonic acid 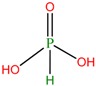	Used as fertilizer and fungicide. Also, used in the production of broad specific antibody for pesticides	[37,38]
SP8	Methylglyoxal 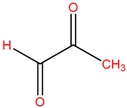	Used in healing diabetic ulcer and anti-bacterial activity against multi drug resistant bacteria.	[39,40]
SP9	Aziridine 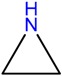	potential therapeutic agents	[41]
SP9	Dimethylamine 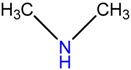	precursor to several industrially significant compounds like DMF, CC etc.	[42]
SP22 SP9	Ethyl acetate 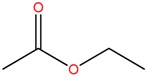	Used as flavoring compounds in wine industry	[43]

## Supplementary Materials

Supplementary materials are available online. Table S1: Diversity indices of collected water samples from Saltpan, Table S2: Relative abundance (%) of majorclasses within the phylum, Table S3: Relative abundance (%) of order, family and genus within the phylum.
