# Supplementary material for: Diversity Analysis and Bioresource Characterization of Halophilic Bacteria Isolated from a South African Saltpan
Source: Molecules. 2017 Apr 20;22(4):657. doi: 10.3390/molecules22040657 (PMC6154464; doi:10.3390/molecules22040657)
Supplement: Supplementary file 1 [file molecules-22-00657-s001.pdf]

Table S1 Diversity indices of collected water samples from Saltpan

Sample ID	Saltpan
Total number of Sequences	3459
Mean Sequences Length (bp)	266±54.8
% of sequence with no relative	23.1
No of OTUs at 0.03	2308
Chao-1 at 0.03	6773.34
Shannon index at 0.03	7.47
No of OTUs at 0.2	683
Chao-1 at 0.2	804.05
Shannon index at 0.2	5.41

Table S2 Relative abundance (%) of majorclasses within the phylum

**Major Classes**	**% of sequences**
Flavobacteriia	40.13
Sphingobacteriia	1.21
Cytophagia	0.27
Cyanobacteria	5.58
Alphaproteobacteria	17.96
Gammaproteobacteria	5.19
Betaproteobacteria	1.05
Deltaproteobacteria	1.05
Actinobacteria	15.86
Planctomycetia	9.72
Deinococci	0.44
Acidobacteria_Gp1	0.16
Acidobacteria_Gp4	0.05
Acidobacteria_Gp3	0.11
Bacilli	0.44
Clostridia	0.38
Erysipelotrichia	0.11
Anaerolineae	0.11
Ktedonobacteria	0.05
Spartobacteria	0.05

Table S3 : Relative abundance (%) of order, family and genus within the phylum

Order	%	Family	%	Genus	%
Flavobacteriales	44.37653	Flavobacteriaceae	40.53651	Salinibacter	4.022989
Sphingobacteriales	1.344743	Rhodothermaceae	1.415797	Salisaeta	0.287356
Cytophagales	0.305623	Chitinophagaceae	0.223547	Segetibacter	0.287356
Rhodobacterales	10.45232	Cytophagaceae	0.149031	GpVII	26.72414
Rhodospirillales	4.645477	Family VII	6.929955	Rubellimicrobium	1.724138
Rhizobiales	2.200489	Rhodobacteraceae	12.74218	Paracoccus	0.287356
Alphaproteobacteria_incertae_sedis	0.061125	Rhodospirillaceae	3.874814	Amylibacter	0.574713
Sphingomonadales	0.183374	Acetobacteraceae	1.490313	Rhodovibrio	13.21839
Caulobacterales	0.061125	Methylobacteriaceae	1.043219	Skermanella	1.149425
Chromatiales	4.217604	Bradyrhizobiaceae	0.298063	Craurococcus	0.574713
Oceanospirillales	0.611247	Beijerinckiaceae	0.149031	Roseomonas	0.287356
Gammaproteobacteria_incertae_sedis	0.061125	Rhizobiaceae	0.074516	Staphylococcus	0.287356
Pseudomonadales	0.061125	Sphingomonadaceae	0.223547	Microvirga	2.873563
Burkholderiales	0.91687	Hyphomonadaceae	0.074516	Methylobacterium	0.862069
Hydrogenophilales	0.061125	Ectothiorhodospiraceae	4.918033	Afipia	0.287356
Myxococcales	1.100244	Halothiobacillaceae	0.074516	Geminicoccus	0.287356
Desulfovibrionales	0.061125	Oceanospirillales_incertae_sedis	0.298063	Novosphingobium	0.287356
Actinomycetales	16.25917	Halomonadaceae	0.149031	Aquisalimonas	0.287356
Rubrobacterales	0.122249	Pseudomonadaceae	0.074516	Spiribacter	9.195402
Solirubrobacterales	0.427873	Oxalobacteraceae	0.819672	Arhodomonas	0.287356
Acidimicrobiales	0.061125	Burkholderiales_incertae_sedis	0.074516	Salicola	1.149425
Nitriliruptorales	0.061125	Comamonadaceae	0.074516	Halovibrio	0.574713

Planctomycetales	10.6357	Hydrogenophilaceae	0.074516	Thiohalorhabdus	0.287356
Deinococcales	0.488998	Polyangiaceae	0.298063	Pseudomonas	0.287356
Bacillales	0.488998	Myxococcaceae	0.149031	Massilia	0.287356
Halanaerobiales	0.244499	Kofleriaceae	0.074516	Noviherbaspirillum	0.574713
Clostridiales	0.183374	Desulfohalobiaceae	0.074516	Variovorax	0.287356
Erysipelotrichales	0.122249	Propionibacteriaceae	1.117735	Thiobacillus	0.287356
Anaerolineales	0.122249	Nocardioidaceae	0.670641	Myxococcus	0.574713
Ktedonobacterales	0.061125	Microbacteriaceae	1.043219	Kofleria	0.287356
		Bogoriellaceae	0.074516	Desulfohalobium	0.287356
		Micrococcaceae	0.52161	Friedmanniella	1.724138
		Intrasporangiaceae	0.074516	Nocardioides	0.287356
		Cellulomonadaceae	0.074516	Marmoricola	1.436782
		Micromonosporaceae	1.19225	Aeromicrobium	0.287356
		Nocardiopsaceae	0.074516	Amnibacterium	0.287356
		Pseudonocardiaceae	0.149031	Agrococcus	0.574713
		Geodermatophilaceae	3.204173	Georgenia	0.287356
		Cryptosporangiaceae	0.074516	Citricoccus	0.862069
		Nakamurellaceae	0.074516	Arthrobacter	0.574713
		Kineosporiaceae	0.223547	Cellulomonas	0.287356
		Streptomycetaceae	0.074516	Blastococcus	2.011494
		Nocardiaceae	0.149031	Modestobacter	2.873563
		Mycobacteriaceae	0.074516	Geodermatophilus	1.724138
		Rubrobacteraceae	0.149031	Cryptosporangium	0.287356
		Solirubrobacteraceae	0.074516	Motilibacter	0.287356
		Patulibacteraceae	0.074516	Nakamurella	0.287356

Nitriliruptoraceae	0.074516	Angustibacter	0.574713
Planctomycetaceae	12.96572	Streptomyces	0.287356
Trueperaceae	0.596125	Gordonia	0.287356
Halobacteroidaceae	0.298063	Mycobacterium	0.287356
Lachnospiraceae	0.074516	Rubrobacter	0.574713
Peptostreptococcaceae	0.074516	Solirubrobacter	0.287356
Erysipelotrichaceae	0.149031	Patulibacter	0.287356
Anaerolineaceae	0.149031	Nitriliruptor	0.287356
Thermosporotrichaceae	0.074516	Singulisphaera	6.321839
		Gemmata	0.574713
		Aquisphaera	3.448276
		Gimesia	0.287356
		Isosphaera	0.287356
		Truepera	2.298851
		Turicibacter	0.574713
		Anaerolinea	0.287356
		Thermosporothrix	0.287356
